# Caregiver approaches, resiliencies, and experiences raising individuals with fetal alcohol spectrum disorder: A study protocol paper

**DOI:** 10.1371/journal.pone.0312692

**Published:** 2024-12-03

**Authors:** Katherine Flannigan, Devon C. Edwards, Dorothy Reid, Audrey McFarlane, Jacqueline Pei

**Affiliations:** 1 Canada Fetal Alcohol Spectrum Disorder Research Network, Vancouver, BC, Canada; 2 Educational Psychology, University of Alberta, Edmonton, AB, Canada; University of Calgary, CANADA

## Abstract

Fetal alcohol spectrum disorder (FASD) is a complex neurodevelopmental disability characterized by a range of brain- and body-based difficulties which, when left unsupported, can lead to experiences of significant adversity across the lifespan. Caregivers of individuals with FASD play a critical role in advocating and supporting healthy outcomes for individuals with FASD, and most caregiver research to date has been focused on stressors and challenges. Very few studies have been conducted to systematically capture the *full* experience of caring for someone with FASD across the lifespan, including perspectives, concerns, as well as strengths and successes of caregivers and their families. Collaborative research with individuals with living experience is essential for understanding needs and supporting healthy outcomes for individuals with FASD and their families, and caregivers are in a unique and important position to provide perspectives and share living expertise. Therefore, the current study was developed collaboratively with caregivers and researchers to capture the many aspects of caregivers’ contexts, concerns, needs, and successes in raising individuals with FASD. In this study protocol paper, we describe the rationale, development, design, and anticipated impacts of this research. The goal of this paper is to share information about why and how this study is being done, and potentially guide other teams in developing similar projects to better understand caregivers’ experiences, needs, and successes. Documenting and giving voice to the breadth and depth of caregiver experiences will help us to tailor services and supports, develop resources, stimulate knowledge translation based in resilience and protective factors, guide future studies, and inform evidence-based policy initiatives.

## Introduction

Fetal alcohol spectrum disorder (FASD) is a complex neurodevelopmental disability caused by prenatal alcohol exposure (PAE) which impacts an estimated 4 to 8% of the general Western population [1, 2]. Individuals with FASD experience significant brain- and body-based differences in cognitive, behavioural, social-emotional, and adaptive functioning requiring comprehensive intervention and support [3]. People with FASD also have notable strengths and abilities and can achieve healthy outcomes when provided with appropriate support for navigating day-to-day life [4]. Without these support systems in place, individuals with FASD can face significant adverse life experiences, including trouble with school and employment, housing instability, mental health needs, substance use, and legal system contact [5, 6].

Parents and caregivers of individuals with FASD hold substantial responsibility in responding to diverse and changing family needs, navigating systems, and advocating for support across the lifespan [7, 8]. Individuals with FASD often need varying levels of support with day-to-day functioning, even as they progress into adulthood [5, 9, 10]. Caregivers have been described as “change agents” in outcomes for individuals with FASD [11] and are often involved in interventions to support wellbeing for those with FASD [12]. High quality and nurturing caregiving environments are among the strongest protective factors for individuals with FASD [6], highlighting the interconnectedness and interdependency between individuals with FASD and their caregivers.

### Caregiver wellbeing

Caring for individuals with FASD comes with challenges and successes that are unique and important to recognize. In recent years, researchers increasingly show that caregivers of individuals with FASD experience high levels of stress and often feel overwhelmed [13–16]. Caregivers who spend more of their time caring for individuals with FASD report lower levels of wellbeing and satisfaction with the supports they receive [14]. Caregivers describe significant concerns about their relationships with family, friends, and the individuals for whom they provide care; the safety and wellbeing of their family members with FASD; their ability to keep up with family needs; their struggles with self-care and meeting their own needs; and the limited access to adequate care they have for their family members with FASD [14, 15, 17–24]. Many caregivers also describe experiencing guilt, regret, grief and loss, and fear about the future, and express feeling misunderstood, judged, blamed, and socially isolated [7, 8, 17, 22, 25–28]. Higher levels of shame, and lower levels of self-compassion and sense of competence as caregivers may further contribute to stress [13, 22], all of which can influence family functioning, social connections, and marital health [8].

Some caregivers report that the parenting stressors associated with FASD are unique [22]. There is preliminary evidence that the challenges of raising an individual with FASD may differ from those associated with parenting children with other disabilities. For example, compared with parents of children with other disabilities, caregivers of individuals with FASD in some studies have been reported to experience uniquely high levels of parenting stress [29] and a greater likelihood of depression and other challenges with mental health [30]. Similarly, parents of children with FASD have expressed distinct concerns and expectations for the future compared with parents of children with autism [28]. Underlying some of these differences may be challenges related to the unique experiences of responding to behaviour and temperament differences of children with FASD, tensions in the parent-child relationship that are unique to FASD [31], limited understanding of FASD among professionals, and a lack of availability of FASD-informed systems and supports for families [32]. Many caregivers report a constant need to be advocates for their family and to “fight” to find support to meet the unique needs of their children [22, 32, 33].

The experiences and stressors of caregivers may vary depending on family demographics, such as the age of their loved one(s) with FASD, caregiver’s life stage, parenting role (i.e., biological, adoptive, foster), family income, access to resources, residential location, and cultural identity [14, 18, 34, 35]. For instance, caregivers who support adolescents with FASD express more concerns than those who support younger children [14], and older caregivers may experience higher levels of stress than those who are younger [22]. Biological parents and Indigenous parents may have especially pronounced experiences of guilt, stigma, marginalization, vulnerability, and oppression [13, 24, 32, 34]. These unique experiences speak to the need for a more nuanced and holistic understanding of how the caregiver experience may differ across families and circumstances.

### Supports and strengths

Researchers consistently report that caregivers desire connections with other caregivers and families, better collaboration and multi-disciplinary support across systems, and more recognition of FASD and knowledge of FASD-related needs at the public and policy levels [7, 14, 23, 36]. Moreover, caregivers emphasize the value of early intervention and the need for respite [23]. However, limited knowledge of FASD among service providers and the public creates a significant barrier to accessing adequate support [19, 33, 37]. This can lead many caregivers to feel ignored or blamed for challenging behaviours exhibited by their children [17, 22]. Importantly, both formal (e.g., service providers and support groups) and informal (e.g., friends and family) supports can help caregivers to manage the experience of raising an individual with FASD [26, 38]. Practicing self-care and adopting an attitude of self-compassion may also help to promote resilience in caregivers, as these approaches may lower levels of distress [13], improve parenting satisfaction, and increase family needs being met [15].

Caregivers of individuals with FASD possess significant strengths in their ability to adapt for their families [34, 39] and to respond with patience, understanding, and flexibility to the needs of their children with FASD [38]. Caregiver education and advocacy efforts can be supported when parents seek information and learn about FASD, which can increase understanding of their children and of how best to parent and meet the needs of their family [39]. Importantly, an increased understanding of FASD can pave the way for more effective parenting strategies [7], and practical approaches have been described by caregivers to help navigate the daily needs and challenges of their families [17, 38, 39].

Caregiver strength is also evident in their expressed hopes for the future of their family members with FASD, with meaningful lives rooted in healthy relationships, community connections, and long-term supports [17, 18, 40]. Caregivers often describe the joy, appreciation, and pride they feel for their children [7, 8, 17, 22, 25, 26, 33]. For many caregivers, the experience of raising an individual with FASD has helped them to view challenges as opportunities, recognize their children’s strengths and gifts, and acknowledge the contributions and fulfillment that their children bring to their lives [7, 39, 41].

### Rationale for the current study

Despite our knowledge about caregiver challenges and the small but growing literature on caregiver strengths and intervention possibilities, important research gaps have been identified. For example, researchers have called for more work on how caregivers’ experiences change as they age, their interactions with different systems (e.g., education, justice) [14], and how sociodemographic factors may be related to caregiver experiences and wellbeing [13]. There are also calls for more research on the root causes of parenting stress for caregivers of individuals with FASD [28], and the potential differences in caregiver experiences of raising an adult (versus child) with FASD [7] as well as across the spectrum of needs for children and adults with FASD [21]. Moreover, very little is known about the specific influence or differences between parenting roles (i.e., biological, adoptive, foster), the gendered experiences of parenting an individual with FASD, or how/whether experiences differ for caregivers raising their grandchildren or great-grandchildren with FASD [30, 39]. There is a need for more diverse participant samples of caregivers beyond adoptive mothers and parents who are predominantly middle-aged and White [13, 42]. More longitudinal research is also needed to better understand the relationships and interactions between factors that influence caregiver wellbeing [15].

## Materials and methods

This protocol paper describes the comprehensive study, *caregiver approaches*, *resiliencies*, *and experiences (CARE) raising individuals with FASD across the lifespan* (the “CARE study”). The broad aim of the CARE study is to capture a breadth of information from caregivers of individuals with FASD across Canada and globally. Study objectives are to: 1) document the many aspects of caregivers’ context, concerns, family functioning, and needs for supporting individuals with FASD; 2) formally record the shared experiences of the caregiving community; 3) give voice to the breadth and depth of caregiver experiences to substantiate research questions, build grants, and guide future studies; 4) capture information that will help us to better tailor services and supports for caregivers; 5) develop informed resources and stimulate knowledge translation for caregivers; and 6) identify and better support caregiver strengths, resiliencies, and protective factors.

More specifically, to address some of the gaps identified in the FASD caregiver literature, the study is guided by the following research questions:

- In what ways do caregivers’ experiences and needs differ based on demographic characteristics (e.g., geographic region, gender, age, marital status, caregiving role [e.g., biological, foster, adoptive], generation [e.g., parent, grandparent, great-grandparent])?
- How do caregivers describe their health and wellbeing, and what support strategies are perceived to be most effective in promoting health and wellbeing? How do caregivers of individuals with FASD describe their skills, strengths, and successes?
- What impacts do major life transitions, changes, and losses (including the COVID-19 pandemic) have on caregivers and families?
- What assessment and intervention services are most commonly accessed by caregivers to support their individuals with FASD, and which ones are perceived as most/least helpful? What are the barriers and facilitators to service access?
- How do caregivers characterize the physical, mental health, social, and behavioural strengths and needs of their individuals with FASD, and in what ways do these strengths and needs impact families?
- How do caregivers describe the educational and employment experiences of their individuals with FASD, including needs and successes?
- What are the future hopes, worries, and needs of caregivers of individuals with FASD?
- How do caregivers’ needs, experiences, and perspectives change over time? Are certain sociodemographic factors associated with caregiver trajectories over time?

Given the exploratory nature of this study, no hypotheses have been established. The goal of this protocol paper is to share information about why and how this research is being conducted, and potentially guide other teams in developing similar work.

### Design and setting

The CARE study is a community-based initiative directly inspired and informed by conversations within the FASD community about caregiver priorities, needs, and knowledge gaps. The conceptualization and design of the study were done in partnership between Canada FASD Research Network (CanFASD) Family Advisory Committee, staff, and researchers. The result of this collaboration is a comprehensive, longitudinal online survey collected and managed using REDCap electronic data capture tools [43, 44] housed at the University of Alberta. The survey was launched September 9, 2021, and data collection is ongoing. Participation is open to caregivers worldwide, and although data has thus far been collected online only, there are also paper and phone options for caregivers who do not have web access.

### Participants

All caregivers of individuals with FASD, of any age, from anywhere in the world, who can read and write in English, are eligible to participate in this study. Caregivers do not have to provide formal documentation of their child or adult’s FASD diagnosis to participate, but must self-identify as a caregiver of someone with FASD. One goal of the CARE study is to capture and document the caregiver experience as broadly and comprehensively as possible. Therefore, we hope to recruit participants from an array of sociodemographic backgrounds and geographic regions across Canada and internationally.

The CARE study is an ongoing, longitudinal study with no anticipated end date. A total sample size has not been calculated, though we aim to recruit a minimum of 500 participants for a sample size sufficient to represent a diverse range of experiences, identify trends across demographic characteristics and family circumstances, and optimize study generalizability. We did not conduct an a priori power analysis but referred to recommendations in the literature regarding minimum sample sizes (~300 to 500) for relatively complex statistical modeling that may be used in this study, such as growth mixture modeling [45].

To recruit participants for the CARE study, we have primarily used convenience and self-selection sampling. When the study was first launched, an online link to the survey was distributed on social media platforms and through various FASD research and community networks and events worldwide. Since then, we continue to recruit through newsletters, social media posts, conference and webinar presentations, and ongoing communication with participants. To participate, interested individuals click on the link provided in recruitment materials and are redirected to the survey, where details about research purpose, procedures, confidentiality, and consent are presented on the first page. The information page indicates that completion and submission of the survey signify an individual’s consent to participate. For participants who complete the survey over the phone, verbal consent will be obtained by a member of the research team before beginning the survey.

As of January 2024, 248 individuals initiated the survey, and 121 completed it in full. The mean age of participants was 54 years (range 25 to 82), and 94% identified as women. Most caregivers (68%) were living in Canada at the time of survey completion. The largest proportion of respondents (59%) were adoptive parents, followed by biological parents or other family members (27%).

### Survey structure and content

The survey is organized into three large sections and requires approximately two hours to complete in full (see Table 1 for more detail). Section 1: *About You* includes items that capture information about caregiver characteristics, family structure and dynamics, and scope of caregiving role. Section 2: *Your Wisdom about People with FASD* contains items that solicit caregiver perspectives on how their loved ones with FASD experience different domains of life and how these experiences impact the caregiver and family. In Section 3: *Final Thoughts*, caregivers are asked to share “words of wisdom” they would offer to a new caregiver and note any other challenges or successes not covered in the survey. The comprehensive nature of the survey allows us to consider relationships between the caregiver, the individual(s) with FASD for whom they care, the broader family, and the systems within which the family functions. This will help us to understand the holistic dynamics of the caregiving experience where multiple domains are interconnected in their impacts across many aspects of life.

**Table 1:** Survey sections and domains explored.

Sections	Domains
*1. About You*	• Sociodemographic characteristics • Family structure and scope of caregiving role • Caregiver health and wellbeing • Family cohesion, stability, and major transitions • Caregiver strategies, resources, and supports
*2 Your Wisdom about People with FASD*	• Early adversity • Physical and mental health • Substance use • Medication • Assessment and therapeutic services • Social and adaptive functioning • Sexual development • Legal system involvement • School and work experiences • Impacts of COVID-19 • Hopes, worries, and expectations for the future
*3. Final Thoughts*	• Advice for new caregivers
*Self-Care* (embedded throughout)	• Guided deep breathing • Inspirational quotes and poems • Short and cost-free self-care ideas • Resources to connect with other caregivers • Grounding activity • Positive affirmations • Gratitude and self-compassion practices

Notably, specific and individual data will not be collected about people for whom participants provide care. All items in survey Section 2: *Your Wisdom about People with FASD* are framed so that caregivers reflect on broad experiences with *all* individuals with FASD who have *ever* been in their care. This decision was made because many caregivers have multiple children and adults with FASD, and repeating the same questions for every dependent was deemed to be overly burdensome. Moreover, because the focus of the CARE study is on the caregiver, we decided that collecting a high-level snapshot of general perspectives and experiences related to raising a person with FASD overall was most in line with our research purpose.

The development of survey items and structure was guided by the existing literature in several ways. First, items related to health and wellbeing (Section 1: *About You*) were inspired by existing validated measures, including the World Health Organization’s Quality of Life scale, Frisch and colleagues’ Quality of Life Inventory, Sears and colleagues’ Well-Being 5, and Felitti et al.’s [46] adverse childhood experiences questionnaire. Questions about the physical, mental health, social, behavioural, and educational/employment needs and experiences of individuals with FASD (Section 2: *Your Wisdom about People with FASD*) were based on Pei and colleagues’ intervention framework, Towards Healthy Outcomes, which was first developed in 2018 and recently updated through collaboration with caregivers and others with living experience. Additional questions, especially open-ended items designed to fill gaps in the existing FASD literature, were developed collaboratively with CanFASD Family Advisory Committee and researchers with cross-disciplinary expertise related to FASD prevention, intervention, and diagnosis, and training in psychology, social work, medicine, and the criminal legal system.

Although most of the survey is comprised of Likert, dichotomous (yes/no), or multiple-choice style questions, several items are open-ended. For example, in the social and adaptive functioning domain, participants are asked, “*What are the greatest social strengths of your child(ren)/adult(s) with FASD*?” In addition, caregivers are asked a series of nine open-ended questions about their hopes, worries, and needs for the future, such as “*What would you need to make the future as successful as possible*?” These questions are designed to collect qualitative information about caregivers’ contexts, experiences, and perspectives.

#### Special considerations

In developing this study, we recognized that the comprehensive and personal nature of the study could place a significant burden on caregivers asked to share their stories. Therefore, we structured the survey so that it may be completed in small portions (10–15 minutes or shorter each) which can be saved and returned to later if breaks are needed. In addition, we embedded short self-care practices throughout the survey to offer caregivers time to reflect, care for themselves in moments of potential discomfort, and possibly learn new coping strategies for later use. Several of these strategies were derived from evidence-based supports identified in the FASD literature (e.g., self-care and self-compassion).

### Participant engagement

Another priority identified during the conceptualization of this research was to remain connected with participants and offer regular feedback. Thus, biannual updates in the form of two-page infographics are developed and disseminated to all caregivers who opt in to receive project feedback. Topics for updates are chosen by the research team based on relevance to current events and/or the time of year (e.g., emerging findings related to school experiences were shared in a “back to school” edition in fall 2022). No financial compensation or incentive is provided to participants. However, as a gesture of appreciation, caregiver responses to the “words of wisdom” survey item will be compiled and translated into a resource to be shared with participants and disseminated more widely to other caregivers of individuals with FASD.

### Survey evolution and participant follow-Up

Because this research is intended to be ongoing and longitudinal, the survey is reviewed regularly, and content will be updated as new evidence, questions, or priorities emerge. Specifically, the study team plans to engage in a full review of the survey every three years. Revisions may include reordering items and/or domains, removing items that have yielded few responses, adding new self-care activities and resources, and creating new items based on emerging findings from the survey and the broader literature using measures aligned with our research questions (e.g., the Caregiver Reaction Assessment Scale, Perceived Stress Scale, Parent Wellbeing Scale, etc.). Between each review cycle (i.e., 18 months after each revision), the research team will seek feedback from participants who consented to be contacted about the study. Feedback will be collected on the perceived helpfulness of study updates; specific findings participants would like to see included in future updates; topic areas participants would like to see added in future iterations of the survey; and any strengths, successes, or helpful strategies that caregivers would like to share.

**Fig 1:**
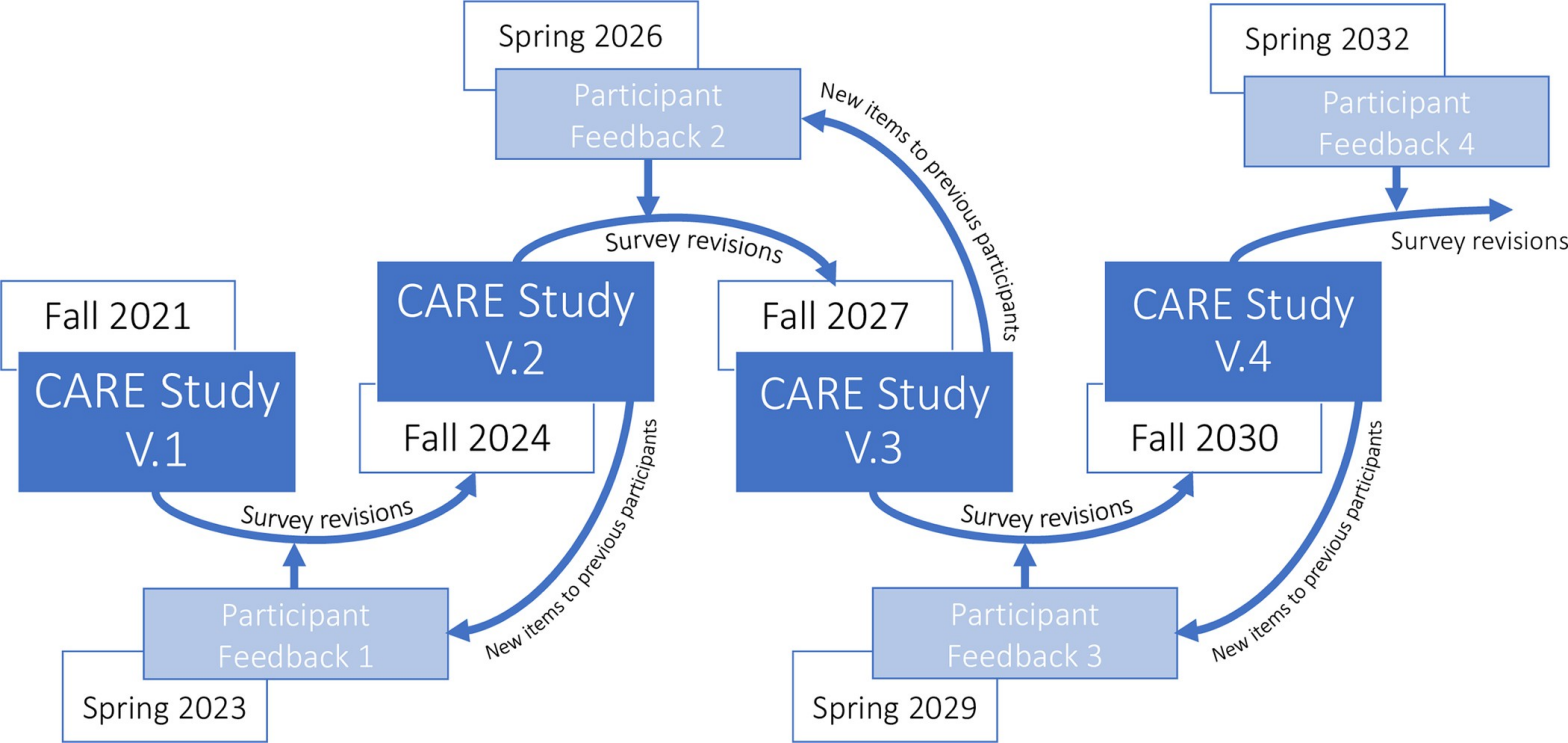
CARE study evolution and timeline.

Once the second iteration of items (“CARE 2.0”) has been finalized (anticipated in fall 2024), caregivers who consented to being contacted will be invited to complete the revised survey. CARE 2.0 will include longitudinal follow-up questions based on the first iteration of the survey (“CARE 1.0”), as well as new items that have been added as part of survey evolution. In this way, there will be three distinct groups of data: 1) “baseline” data collected initially through CARE 1.0; 2) longitudinal data collected through CARE 2.0 based on baseline questions; and 3) new data collected only through CARE 2.0. This process will be followed for all participants, regardless of when they are first recruited. Follow-up data will be collected from consenting participants every three years, coinciding with survey revisions/updates (see Fig 1 below for approximate timelines).

#### Longitudinal data

Longitudinal data will be collected every three years to address our research questions about the ways in which caregiver needs, experiences, and perspectives change over time, and to identify potential trajectories in these experiences. Outcome variables of interest include caregiver wellbeing, coping strategies, family cohesion and stability, and access to services. Potential covariates include age, gender, and ethnicity (of both caregiver and individual[s] cared for), caregiving role, location, socioeconomic status, marital status, and employment status. Longitudinal changes will be examined using items already present in the survey; that is, items from baseline (CARE 1.0) will be repeated at follow-up (CARE 2.0) to assess change in these variables over time. We may also include open-ended questions for participants to qualitatively describe any changes they have experienced since their previous survey response. Because we are not collecting individual data about participants’ dependents, we will not be exploring changes over time among the children and adults with FASD for whom participants provide care. Rather, longitudinal analysis will be focused on new experiences or changes that caregivers may have had between data collection timepoints.

At the time of initial survey completion, participants who agree to be contacted for future research are asked to generate a unique identification number. For longitudinal follow-up, researchers will send out invitations to participants who consented to being contacted, who will be asked to enter their unique ID number so that responses can be linked without personal identification. We have used several strategies to promote retention in the CARE study based on the longitudinal research literature [47, 48] including barrier-reduction (e.g., offering alternate modalities for data collection, minimizing the length of follow-up surveys); follow-up and reminder strategies (e.g., maintaining regular communication, study updates twice/year); and community building and incentives (e.g., offering education and self-care strategies, the words of wisdom resource).

### Data management and dissemination

This study has received approval from the University of Alberta Research Ethics Board (Pro0010934) and data management procedures align with these institutional policies and regulations. Specifically, participant responses are saved anonymously to REDCap, which is locally hosted within the University of Alberta’s Faculty of Medicine and Dentistry’s data centre. When data is to be analysed, raw responses are downloaded to an offline document that is stored on a password-protected and encrypted computer.

Because there is no monetary incentive for completing this survey, we expect that the risk of having bots in the sample is quite low. As well, there are several open-ended items early in the survey which are helpful for detecting bots, and we collect timestamps to monitor how quickly items were answered. We also screen and monitor data on a routine basis and have detected no suspicious responding thus far. Identifying information (name and contact details) are collected and retained *only* for participants who choose to receive resources developed from the study, or those who wish to be contacted for future research. We retain a list of participants who opt in to be contacted about this study and about future research, but information is never linked to survey responses. All identifying information is kept in a secure password-protected file, and stored separately from the survey data so responses cannot be linked to any individual. All materials developed from this study are anonymous except for when a participant explicitly requests to have their name published with the “words of wisdom” document in recognition of their contribution.

Although this study is not intended to generate a public-use dataset, data is available for other researchers to request through a structured process, leaving opportunities for unique research questions and analyses. Researchers who wish to request data for use in their own work must first apply for ethics approval through their institutional Research Ethics Boards for secondary use of data and submit their approved ethics application along with a completed data request form to the study team for consideration. All non-identifying variables collected in the survey are eligible for request, but only those that are explicitly requested and approved will be shared. Applications must align with the ethics and overarching purpose of the overall study. Upon approval of a data request, a study team member will retrieve the requested information and share the raw data through email via a password-protected file.

Emerging findings from this study have been disseminated in several ways, including biannual participant updates, social media posts, conference presentations, and webinars.

### Planned analyses

The CARE study captures a breadth of caregiver experiences across various settings and contexts. Because of the study’s exploratory nature, specific analyses are not yet planned, but we broadly intend to characterize the responding population of caregivers along with their experiences and perspectives across many domains of wellbeing and functioning. The multifaceted nature of the survey data (i.e., quantitative and qualitative) allows for multiple approaches to data analysis. For example, to address our research question regarding demographic trends in caregiver experiences and needs, we may use descriptive analyses to examine frequencies and characterize the study population, as well as inferential statistics such as chi-square tests, multivariate analysis of variance, or logistic regression to examine patterns or trends based on caregiver age, gender, marital status, caregiving role, etc. Approaches such as thematic or narrative analysis may be used for open-ended qualitative data. To manage attrition and missing data, imputation or weighting adjustment methods may be used. Depending on the amount, nature, and quality of longitudinal data, analysis of quantitative data may include repeated measures analysis of variance, generalized estimating equations, growth mixture modelling, or mixed effects regression. Analysis strategies for qualitative data may include case studies, recurrent cross-sectional analysis, or trajectory analysis.

## Discussion

To date, there has been very little research to systematically capture the *full* experience of caring for someone with FASD across the lifespan, including the wide-ranging and unique experiences, most pressing concerns, and strengths and successes of caregivers and their families. There is also a lack of comprehensive research that allow us to explore whether and how caregiver experiences may vary across sociodemographic characteristics and in different parts of the world. Most previous studies related to caregiver experiences have been focused predominantly on the stressors associated with the caregiving role rather than the resiliencies, successes, and strengths. The current study will fill a number of these research gaps. By exploring sociodemographic trends in caregiver experiences, caregiver health and wellbeing, use and impacts of support strategies, barriers and facilitators to service access, future hopes and needs, as well as the ways in which the needs of children and adults with FASD impact caregivers and families, a more holistic understanding of the caregiver experience with be achieved. Findings related to caregiver trajectories and changes in wellbeing and experiences over time will also contribute much needed knowledge of longitudinal trends. Importantly, a strengths-based lens was used in the development of this research which will allow for a more balanced reflection of both the difficulties *and* the successes of individuals with FASD and their caregivers to help reduce stigma and support hope for healthy outcomes.

In developing this study, we prioritized honouring and “giving back” to participants in tangible ways through the inclusion of self-care activities, informing participants with regular study updates, and offering caregivers an opportunity to share what they have learned through “words of wisdom,” as an affirmation of their resilience and a way of making their experience meaningful. Caregiver perspectives, experiences, and stories inherently matter, including both the stressors *and* the successes, and systematic exploration and documentation of living experience is often necessary to stimulate new research and inform practice and policy. Our hope is that this research will generate information that helps us to both maintain critical supports that currently exist, and to inform the development of novel supports for caregivers and their families as an integrated foundation for healthy outcomes.
